# Mesomorphic Properties of an Homologous Series of Thioalkyl-Terminated Azomesogens

**DOI:** 10.3390/ijms12053182

**Published:** 2011-05-16

**Authors:** Uhood J. Al-Hamdani

**Affiliations:** Department of Chemistry, College of Education, Basrah University, P.O. Box 716, Basrah, Iraq; E-Mail: Uhood1959@yahoo.com; Tel.: +009647712616545; Fax: 009641414811

**Keywords:** azo, terminal thioalkyl, Nematic

## Abstract

A new homologous series (ten compounds) of 2-hydroxy azo compounds SR*_n_* (where *n*:1–10) were synthesized. Their structures were elucidated using spectroscopic techniques such as IR (Infrared), ^1^H-NMR as well as elemental analysis. Mesomorphic properties and phase transitions were studied using polarized hot stage optical microscopy and differential scanning calorimetry (DSC), and are discussed as a function of the number of carbon atoms in the thioalkyl chain. It has been found that all compounds in the series are pure nematogens.

## 1. Introduction

**Figure 1.**
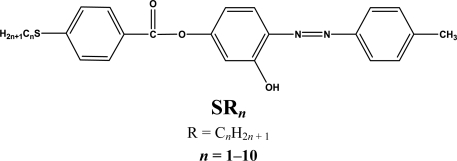
Structures of the studied compounds SR_1_–SR_10_).

Liquid crystalline materials are of great interest for materials science as well as for life science. Their properties can be tuned by appropriate molecular design. Thus, it is well known that the mesomorphic properties of calamitic liquid crystals can largely be influenced by the structural variation in terminal substituents, like alkyl, alkyloxy and thioalkyl groups [1–5]. This can influence the melting points and mesophase types, *etc.* [6–10]. Numerous studies have been carried out in recent years of the effects of changes in molecular framework on the incidence and stability of azo ester liquid crystal phase [11–14]. However, a literature survey indicates that liquid crystalline azo esters with thioalkyl chains comprising different kinds of terminal groups are very rare, so, in the present work, we have prepared ten members (Figure 1) of homologous series of (SR*_n_*) in order to establish the effect of introducing sulfur atom instead of oxygen atom with the increase in the terminal thioalkyl chain on liquid crystalline properties.

## 2. Result and Discussion

**Figure 2.**
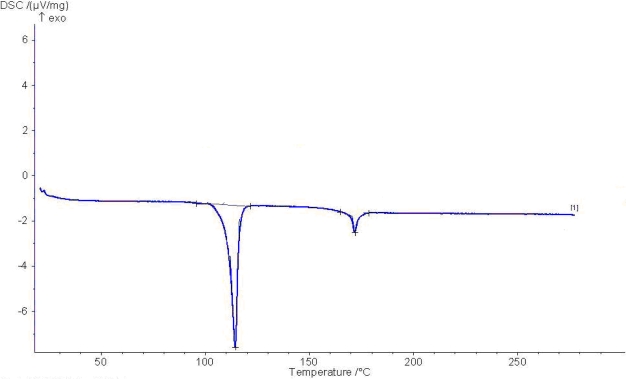
DSC thermogram for E-SR_8_.

The DSC scans carried out on these compounds show transitions at temperatures which are in agreement with those obtained by optical microscopy. The phase transition temperatures of all compounds are given in Table 1. All the studied azo compounds exhibit enantiotropic mesogenic behavior. All members of the series show Nematic phase (N) only (Figure 2).

**Figure 3.**
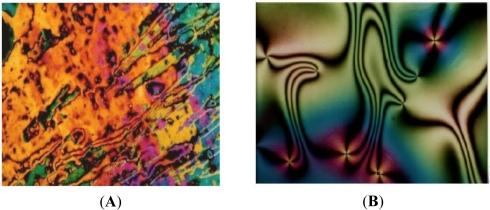
(**A**) Marble texture for Nematic phase in heating at 150 °C for SR_1_; (**B**) Schlieren texture for Nematic phase in cooling at 234 °C for SR_1_.

The Nematic phase reflects the marbled texture on heating and the schlieren texture on cooling (Figure 3).

**Figure 4.**
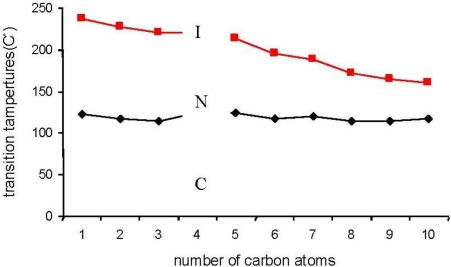
Phase transition temperatures as a function of the thioalkyl chain length for the SR*_n_* series (Black: **C→N**; Red: **N→I**; C: Crysta l; N: Nematic; I: Isotropic).

A plot of transition temperatures against the number of carbon atoms, *n*, in the thioalkyl chain for the studied compounds (SR*_n_*) is given in Figure 4. The plot shows typical mesomorphic trends. The Nematic-isotropic transition temperature curve is a falling one throughout the homologous series. At longer thioalkyl chain lengths, the Nematic phase stability range decreases [15,16].

We did not notice the phenomenon of odd–even during the study of the transition temperatures for the prepared compounds. This result coincides with many results from other studies [15,17].

The appearance of this phenomenon in the mesomorphic compounds depends on the molecular structure [5].

Increase in the alkyl chain length should have two effects:

1. Increases the intermolecular attractions between the sides of the molecules, because of the polarizability of each added methylene group;
2. Decreases the intermolecular terminal attractions because of the increasing separation of the molecules containing the dipolar units.

Increase in the alkyl chain length should therefore increase the ratio of the lateral to the terminal attractions between the molecules, so making the probability greater that the layer arrangement will persist after melting when the terminal attractions are weakened. Smectic properties are therefore most likely to be observed in the long chain members of an homologous series of mesomorphic compounds. A common pattern of behavior is that the lower homologous are Nematic, the middle members exhibit a smectic mesophase followed by a nematic and the long chain members are purely smectic. However, our study of the effect of the chain length on the mesomeric properties of the prepared compounds shows the formation of nematic phase only, which indicate that the effect of the terminal attraction forces are higher than the lateral attraction forces which leads to the formation of nematic phase [18,19].

The formation of a high proportion of this kind of attraction forces is due to the presence of sulfur atoms, which are large, and prevent the aggregation of the molecules to form the layer shape and the appearance of the smectic phase. These results were also found in many other studies for compounds which contain sulfur atoms [20]. The compound which have the similar molecular structure with our compounds but substituted by alkyloxy group exhibit the nematic phase as well as smectic phase due to the difference between the oxygen and sulfur in volume [12].

## 3. Experimental

### 3.1. General

Infrared spectra were recorded as KBr pellets on a Buck-M500 spectrometer. ^1^H-NMR and ^13^C-NMR spectra were recorded on Gemini-200 using CDCl_3_ as a solvent and TMS (TetraMethylSilane (CH_3_)_4_SI) as internal standard. Elemental analysis was performed on Euro Vectro EA 3000A. The phase transitions were observed with a Leitz Laborlux 12 Pol optical microscope with polarized light in conjunction with a leitz 350 hot stage equipped with a Vario-Orthomat camera of transition temperatures were made using a Shimdzu 24 DSC-50 differential scanning calorimeter with a heating rate of 10 °C min^−1^.

### 3.2. Synthesis of Azo Compound A-OH

#### 3.2.1. Diazotization of p-Toluidine

**Scheme 1.**
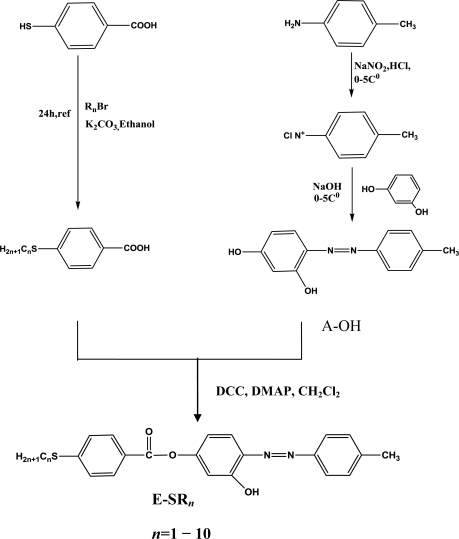
Synthesis steps of the prepared compounds.

A solution of p-Toluidine (10 mmol) in (8 mL 3MHCl) was heated gently, 10 mL of water was added in order to dissolve the solid. The mixture was cooled to 0 °C in an ice bath with stirring. Some solid may precipitate, but the reaction will still work well if it is stirred. 10 mL of freshly prepared 1M sodium nitrite solution was then added slowly with stirring. The rate of addition was adjusted so that the temperature of the solutions remained below 10 °C [14]. The solution was kept in an ice bath and proceeds immediately to the next step (Scheme 1).

#### 3.2.2. Coupling with Phenol

A solution of one of the substituted phenol (10 mmol) in 20 mL of 1 M NaOH, was prepared and cooled in an ice bath. The diazonium salt (step 1) then added slowly with stirring to the phenol solution. The reaction mixture then stands in the ice bath for at least 15 minutes until the crystallization is completed (a colored solid). The pH of the solution was adjusted with dilute HCl or NaOH solutions (0.1 M) in order to induce precipitation. The orange azo dye was then collected and washed in cold water [14].

### 3.3. Synthesis of Esters E-SR_*n*_

Solutions of 4-*n*-thioalkyl benzoic acid (10 mmol), (10 mmol) 1,3-dicyclohexylcarbodiimide (DCC) (55 mmol) in 50 mL dry dichloromethane along with solid 4-dimethyl amino pyridine (DMAP) as catalyst (2.5 mmol) were magnetically stirred at room temperature for 12 h. The byproduct (dicyclohexyl urea) was filtered off under suction and the solvent was removed on rot vapor. The crude product was recrystallized from hot solution of ethanol [21].

*4-*((*4-tolylphenyl*)*diazenyl*)*phenol* (**A-OH**): yield% 45, m.p.: 192–195 °C, IR cm^−1^: 3260 (broad OH), 1600 (C=C), Elemental analysis calculated for C_13_H_12_O_2_N_2_: %C 68.42, %H 5.26, %N 12.28. found: %C68.72, %H 5.27, %N 12.33.

*3-hydroxy-4-*((*p-tolyldizenyl*)*methyl*)*phenyl-4-*(*methylthio*)*benzoate* (**E-SR_1_**): Orange solid; yield% 40; ^1^H-NMR (CDCl_3_): 2.43 (S, 3H, CH_3_), 2.98 (S, 3H, SCH_3_), 6.90–8.10 (m, 11H, Ar-H), 13.3 (S, 1H, OH); IR cm^−1^: 1727 (C=O), 1591–1458 (C=C); Elemental analysis calculated for C_21_H_18_O_3_N_2_S: %C 66.66, %H 4.76, %N7.40, %S 8.46. found: %C 66.96, %H 4.77, %N 7.51, %S 8.58.

*3-hydroxy-4-*((*p-tolyldizenyl*)*methyl*)*phenyl-4-ethylthio*)*benzoate* (**E-SR_2_**): Orange solid; yield% 40; ^1^H-NMR (CDCl_3_): ^1^H-NMR (CDCl_3_): 1.07 (t, 3H, CH_3_), 2.43 (S, 3H, CH_3_), 3.00 (q, 2H, SCH_2_), 6.90–8.10 (m, 11H, Ar-H), 13.3(S, 1H, OH); IR cm^−1^: 1730 (C=O), 1452–1604 (C=C); Elemental analysis calculated for C_22_H_20_O_3_N_2_S: %C 67.34, %H 5.10, %N 7.14, %S 8.16. found: %C 67.54, %H 5.12, %N 7.18, %S8.21.

**Figure 5.**
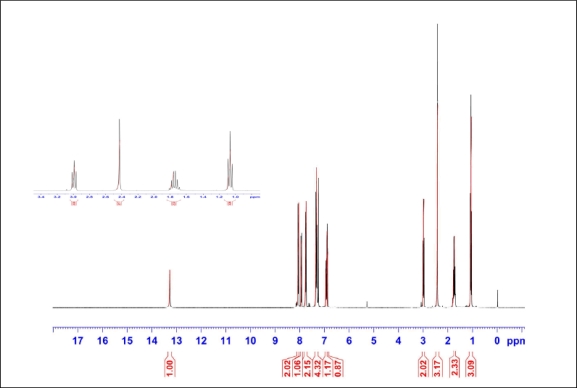
^1^H-NMR for E-SR_3_ with expansion for aliphatic protons.

*3-hydroxy-4-*((*p-tolyldizenyl*)*methyl*)*phenyl-4-propylthio*)*benzoate* (**E-SR_3_**): Orange solid; yield% 43; ^1^H-NMR (CDCl_3_): 1.07 (t, 3H, CH_3_), 1.75 (Hextet, 2H, CH_2_), 2.42 (S, 3H, CH_3_), 2.98 (t, 2H, SCH_2_), 6.90–8.10 (m, 11H, Ar-H), 13.3 (S, 1H, OH) (Figure 5); IR cm^−1^: 1732 (C=O), 1450–1600 C=C); Elemental analysis calculated for C_23_H_22_O_3_N_2_S: %C 67.98, %H 5.41, %N 6.89, %S 7.88. found: %C 68.29, %H 5.51, %N 6.94, %S 7.93.

*3-hydroxy-4-*((*p-tolyldizenyl*)*methyl*)*phenyl-4-butylthio*)*benzoate* (**E-SR_4_**): Orange solid; yield%56; ^1^H-NMR (CDCl_3_): 1.07 (t, 3H, CH_3_), 1.60 (P, 2H, CH_2_), 1.75 (Hextet, 2H, CH_2_), 2.42(S, 3H, CH_3_), 3.00 (t, 2H, SCH_2_), 6.90–8.10 (m, 11H, Ar-H), 13.3(S, 1H, OH), IR cm^−1^: 1725 (C=O), 1456–1590 (C=C); Elemental analysis calculated for C_24_H_24_O_3_N_2_S: %C 68.57, %H 5.71, %N 6.66, %S 7.61. found: %C 68.75 ,%H5.79, %N 6.84, %S 7.72.

*3-hydroxy-4-*((*p-tolyldizenyl*)*methyl*)*phenyl-4-pentylthio*)*benzoate* (**E-SR_5_**): Orange solid; yield%58; ^1^H-NMR (CDCl_3_): 1.07 (t, 3H, CH_3_), 1.75 (P, 2H,CH_2_, 1.60–1.31 (m, 4H, (CH_2_)_2_, 2.42 (S, 3H, CH_3_), 3.00 (t, 2H, SCH_2_), 6.90–8.10 (m, 11H, Ar-H), 13.3(S, 1H, OH); IR cm^−1^: 1721 (C=O), 1450–1596 (C=C); Elemental analysis calculated for C_25_H_26_O_3_N_2_S: %C 69.12, %H 5.99, %N 6.45,%S 7.37 found: %C 69.34, %H 6.07, %N 6.53, %S 7.43.

*3-hydroxy-4-*((*p-tolyldizenyl*)*methyl*)*phenyl-4-hexylthio*)*benzoate* (**E-SR_6_**): Orange solid; yield%60; ^1^H-NMR (CDCl_3_): 1.07 (t, 3H, CH_3_), 1.75 (P, 2H, CH_2_, 1.29–1.31 (m, 6H, (CH_2_)_3_, 2.42 (S, 3H, CH_3_), 3.00 (t, 2H, SCH_2_), 6.90–8.10 (m, 11H, Ar-H), 13.3 (S, 1H, OH); IR cm^−1^: 1728 (C=O) 1455–1600 (C=C); Elemental analysis calculated for C_26_H_28_O_3_N_2_S: %C 69.64, %H 6.25, %N 6.25, %S 7.14. found:%C69.94, %H 6.27, %N 6.29%, S 7.19.

*3-hydroxy-4-*((*p-tolyldizenyl*)*methyl*)*phenyl-4-heptylthio*)*benzoate* (**E-SR_7_**): Orange solid; yield%66; ^1^H-NMR (CDCl_3_): 1.07 (t, 3H, CH_3_), 1.75 (P, 2H, CH_2_, 1.29–1.31 (m, 8H, (CH_2_)_4_), 2.42 (S, 3H, CH_3_), 3.00 (t, 2H, SCH_2_), 6.90–8.10 (m, 11H, Ar-H), 13.3 (S, 1H, OH); IR cm^−1^: 1726 (C=O), 1451–1595 (C=C); Elemental analysis calculated for C_27_H_30_O_3_N_2_S: %C 70.12, %H 6.49, %N 6.06, %S 6.92. found: %C 70.37, %H6.55, %N6.13, %S 6.97.

*3-hydroxy-4-*((*p-tolyldizenyl*)*methyl*)*phenyl-4-octylthio*)*benzoate* (**E-SR_8_**): Orange solid; yield%73; ^1^H-NMR (CDCl_3_): 1.07 (t, 3H, CH_3_), 1.75 (P, 4H, CH_2_, 1.29–1.31(m, 14H, (CH_2_)_5_), 2.42 (S, 3H, CH_3_), 3.00 (t, 2H, SCH_2_), 6.90–8.10 (m, 11H, Ar-H), 13.3 (S,1H,OH); IR cm^−1^: 1748(C=O), 1451–1599 (C=C); Elemental analysis calculated for C_28_H_32_O_3_N_2_S: %C 70.58, %H 6.72, %N 5.88, %S 6.72. found: %C70.76, %H6.77, %N 5.93 %S6.79.

*3-hydroxy-4-*((*p-tolyldizenyl*)*methyl*)*phenyl-4-nonylthio*)*benzoate* (**E-SR_9_**): Orange solid; yield%73; ^1^H-NMR (CDCl_3_): 1.07 (t, 3H, CH_3_), 1.75 (P, 4H, CH_2_, 1.31–1.42 (m, 12H, (CH_2_)_6_), 2.42 (S, 3H, CH_3_), 3.00 (t, 2H, SCH_2_), 6.90–8.10 (m, 11H, Ar-H), 13.3 (S, 1H, OH); IR cm^−1^: 1728 (C=O), 1453–1601 (C=C); Elemental analysis calculated for C_29_H_34_O_3_N_2_S: %C 71.02, %H 6.93, %N 5.71, %S 6.53. found: %C71.24, %H6.98, %N5.78, %S 6.60.

*3-hydroxy-4-*((*p-tolyldizenyl*)*methyl*)*phenyl-4-decylthio*)*benzoate* (**E-SR_10_**): Orange solid; yield%76; ^1^H-NMR (CDCl_3_): 1.07 (t, 3H, CH_3_), 1.75 (P, 4H, CH_2_, 1.29–1.31 (m, 14H, (CH_2_)_7_), 2.42 (S, 3H, CH_3_), 3.00 (t, 2H, SCH_2_), 6.90–8.10 (m, 11H, Ar-H), 13.3 (S, 1H, OH); IR cm^−1^: 1728 (C=O),1452–1600 (C=C); Elemental analysis calculated for C_30_H_36_O_3_N_2_S: %C 71.42, %H 7.14, %N5.55, %S 6.34. found: %C71.71, %H7.18, %N5.61, %S 6.38.

## 4. Conclusions

New azo mesogenic compounds with a thioalkyl chain on the terminal of the benzene ring were synthesized. The study indicates that the length of the thioalkyl chain has an effect on the mesomorphic properties, *i.e.*, the temperature range of the nematic phase and melting points decrease with increasing the chain length, however, the large size of the sulfur atom prevents the smectic phase from appearing.

## Figures and Tables

**Table 1.** The phase transitions temperatures (°C) of compounds and associated enthalpy data (kJ/mol, in parentheses).

**Compound**	**C→N**	**N→I**	**Δ*T*_N_**
SR_1_	123.0 (25.53)	237.0 (1.45)	114.0
SR_2ٍ_	118.0 (26.99)	228.0 (1.31)	110.0
SR_3ٍ_	114.9 (29.66)	220.7 (1.23)	105.8
SR_4ٍ_	124.9 (38.14)	220.0 (1.22)	95.10
SR_5ٍ_	124.4 (39.46)	213.7 (1.11)	89.30
SR_6ٍ_	117.5 (39.95)	194.9 (1.15)	77.40
SR_7ٍ_	119.9 (41.85)	189.0 (1.19)	69.10
SR_8ٍ_	114.5 (42.32)	171.9 (1.16)	57.40
SR_9ٍ_	115.2 (43.46)	165.0 (0.83)	49.80
SR_10ٍ_	117.9 (44.75)	160.2 (1.21)	42.30

C = solid; N = Nematic phase; I = Isotropic. Δ*T*_N_: Thermal range of Nematic phase.
